# Protein and Micronutrient Intake After Two Years of Sapropterin Treatment in PKU

**DOI:** 10.3390/nu18101549

**Published:** 2026-05-13

**Authors:** Ozlem Yilmaz Nas, Catherine Ashmore, Maria Ines Gama, Anne Daly, Sharon Evans, Alex Pinto, Yahya Ozdogan, Anita MacDonald

**Affiliations:** 1 Dietetic Department, Birmingham Women’s and Children’s Hospital, Birmingham B4 6NH, UK; catherine.ashmore@nhs.net (C.A.); maria.gama1@nhs.net (M.I.G.); a.daly3@nhs.net (A.D.); sharon.morris6@nhs.net (S.E.); alex.pinto@nhs.net (A.P.); anita.macdonald@nhs.net (A.M.); 2 Department of Nutrition and Dietetics, Ankara Yildirim Beyazit University, Ankara 06760, Turkey; yozdogan@aybu.edu.tr

**Keywords:** sapropterin, micronutrients, dietary liberalisation, protein substitutes

## Abstract

**Background:** Sapropterin allows dietary liberalisation in responsive individuals with phenylketonuria (PKU), increasing natural protein intake and reducing dependence on protein substitutes (PSs). As PSs provide essential micronutrients, dietary liberalisation may increase the risk of nutritional insufficiency. Evidence describing detailed micronutrient intake in sapropterin-treated children remains limited. **Methods:** This secondary analysis evaluated dietary protein and micronutrient intake after 24 months of sapropterin treatment in 21 responsive children from a prospective longitudinal study. Caregiver-completed three-day food records were analysed for protein, calcium, iron, zinc, vitamin D, and vitamin B_12_, with micronutrient intakes compared with UK dietary reference values (DRVs). **Results:** Mean total protein intake was 75 ± 14 g/day, comprising 30 ± 16 g/day natural protein and 45 ± 21 g/day protein equivalent from PSs; natural protein tolerance ranged from 8 to 66 g/day. PSs contributed most micronutrients: calcium 80%, iron 84%, zinc 87%, vitamin D 96%, and vitamin B_12_ 78%. Median micronutrient intakes exceeded DRVs for most children; however, four had intakes below DRVs, almost exclusively when PSs were reduced or omitted. One child consuming >40 g/day natural protein without PSs had low iron (51%), zinc (90%), and vitamin D (4%) intakes. A non-adherent adolescent had low intakes of calcium (46%), iron (64%), zinc (41%), and vitamin D (60%). Another child receiving 60 g/day protein equivalent from PSs had marginally low vitamin D intake (85%) due to lower fortification. Children maintaining regular PS use met micronutrient requirements. **Conclusions:** After two years of sapropterin treatment, dietary liberalisation increased natural protein intake but did not consistently ensure adequate micronutrient intake. Micronutrient shortfalls were associated with reduced PS use, emphasising the need for careful dietitian-guided adjustment as diets become more flexible.

## 1. Introduction

Phenylketonuria (PKU) has traditionally been managed with a lifelong natural protein intake restriction, supported by low-phenylalanine (Phe) protein substitutes and specialised low-protein foods [1]. This dietary approach remains highly effective for maintaining metabolic control and preventing neurocognitive impairment, yet it is also one of the most restrictive dietary regimens in metabolic medicine. Adherence commonly declines with age, reflecting the cumulative burden of meticulous dietary calculation, limited food choice, and the social and psychological challenges associated with long-term dietary vigilance [2,3,4,5,6]. These difficulties contribute to suboptimal metabolic control in adolescence and adulthood, with implications for executive function, mental health, and quality of life [7,8,9,10,11,12,13,14].

However, the therapeutic landscape for children with PKU is changing with the introduction of oral pharmacological therapies that target residual Phe hydroxylase (PAH) activity. Sapropterin dihydrochloride, a synthetic formulation of tetrahydrobiopterin (BH4), was the first agent to demonstrate clinically meaningful reductions in blood Phe concentrations in responsive individuals, enabling increased natural protein tolerance [15,16]. More recently, sepiapterin, an orally administered precursor of BH4, has shown promise in increasing the proportion of individuals who may benefit from PAH-activator therapy, including some who do not respond to sapropterin [17,18,19].

Higher Phe tolerance allows the gradual inclusion of foods that were previously restricted in the classical PKU diet, including regular cereal products and, in some individuals, animal-derived protein sources such as meat, fish, eggs, and dairy products [20]. This change represents an important nutritional transition, as many of these foods provide not only high-quality protein but also micronutrients, such as iron, vitamin B_12_, calcium, and long-chain polyunsaturated fatty acids, that are limited in the traditional low-Phe diet [21]. However, the extent of dietary liberalisation varies considerably between individuals. Differences in pharmacological responsiveness, baseline Phe tolerance, and long-standing eating behaviours strongly influence the pace and breadth of dietary change. In addition, concerns about metabolic stability, fear of losing control, and uncertainty about how to incorporate higher-protein foods safely often lead to cautious, incremental adjustments rather than full dietary expansion [22,23].

Evidence from both paediatric and adult cohorts indicates that even when metabolic tolerance increases substantially, many individuals continue to rely on familiar low-protein staple foods and introduce higher-protein sources selectively [22,24,25]. This pattern reflects a complex interplay between physiological capacity and behavioural adaptation. For individuals who have adhered to a highly restrictive diet since infancy, food preferences, sensory familiarity, and established routines may limit the uptake of new foods, even when permitted. Moreover, the psychological burden of decades of dietary vigilance can make the transition toward a more liberal diet feel risky or destabilising. As a result, dietary liberalisation often unfolds as a gradual, personalised process rather than a uniform or immediate modification.

These evolving dietary patterns may have important nutritional implications. In the traditional PKU diet, low-Phe protein substitutes provide a major source of essential micronutrients because they are usually fortified with vitamins and minerals to compensate for the limited nutrient profile of very low-protein diets. When intake of protein substitutes decreases following sapropterin or sepiapterin treatment, micronutrient intake may decline if the additional natural foods introduced do not provide sufficient nutritional value [26]. This risk is particularly relevant when dietary expansion focuses on energy-dense but nutrient-poor foods, or when individuals remain hesitant to incorporate nutrient-rich animal-derived products despite increased Phe tolerance.

Evidence from studies of liberalised diets, including sapropterin-treated populations, consistently shows that reducing or discontinuing fortified protein substitutes is frequently associated with intakes below recommended levels for several micronutrients. Reported shortfalls include vitamin D, vitamin B_12_, folate, calcium, iron, iodine, selenium, and zinc [17,21,24,27,28,29]. Assessing nutritional adequacy is also challenging. Biochemical markers do not always reliably reflect dietary intake or early micronutrient insufficiency, particularly for nutrients with tight homeostatic regulation or large body stores [30]. As a result, individuals may appear biochemically replete despite declining dietary intake, delaying recognition of emerging deficiencies.

In children, inadequate intake of key nutrients may impair linear growth, reduce bone mineral accrual, and adversely affect neurodevelopment, rendering the nutritional consequences of dietary liberalisation particularly important to evaluate [1,31]. Studies of individuals following relaxed or unrestricted diets report lower consumption of animal-derived foods, fruits, and vegetables, alongside continued reliance on cereal-based foods and other familiar low-protein staples [24,27]. Such dietary patterns increase the risk of micronutrient insufficiency, and these vulnerabilities arise when reductions in fortified protein substitutes, which typically provide the majority of micronutrient intake in PKU, are not accompanied by the introduction of nutrient-dense foods. Despite these concerns, data describing micronutrient intake specifically in sapropterin-treated children remain limited. Most available studies focus on adults or mixed-age cohorts, and few provide detailed nutrient profiling or longitudinal follow-up. As a result, the extent to which dietary liberalisation in childhood, whether partial or substantial, affects micronutrient adequacy, growth trajectories, or biochemical markers of nutritional status is not yet well characterised.

In our previously published two-year prospective study [20], sapropterin-responsive children demonstrated substantial increases in natural protein intake together with a broader range of food choices, including the introduction of animal-derived foods. The present report examines the dietary protein and micronutrient intake after 24 months of sapropterin therapy in responsive children, providing insight into the nutritional quality of liberalised diets and informing evidence-based dietetic guidance for this population.

## 2. Materials and Methods

### 2.1. Study Design and Participants

This report presents a secondary analysis of children identified as sapropterin-responsive within our previously published prospective longitudinal study conducted at Birmingham Children’s Hospital, United Kingdom (UK). For the present analysis, only participants who met the predefined criteria for sapropterin responsiveness were included. Responsiveness was determined according to the British Inherited Metabolic Disease Group (BIMDG) sapropterin treatment pathway. Full details of the responsiveness testing protocol, study design, and the original inclusion and exclusion criteria have been described previously [20]. Briefly, inclusion criteria were children aged 3–17 years with a confirmed diagnosis of PKU or dihydropteridine reductase (DHPR) deficiency, managed with a Phe-restricted diet; children who had late diagnosis or significant comorbidities unrelated to PKU were excluded. Sapropterin responsiveness was defined as a ≥30% reduction in blood Phe during a standardised 28-day protocol, as per BIMDG guidance. Children who remained on dietary treatment alone and did not commence sapropterin therapy were excluded from the current analysis. For the purposes of this paper, analyses were limited to dietary data collected at 24 months following initiation of sapropterin treatment, and the analysis is therefore primarily descriptive rather than a full longitudinal assessment of nutritional change over time. Baseline dietary data were not reported, as the primary aim was to evaluate protein and micronutrient intake after sapropterin treatment and associated dietary liberalisation.

### 2.2. Data Collection

Demographic and clinical information, including age, sex, and relevant medical history, were obtained from hospital medical records. Dietary intake at 24 months was assessed using 3-day food records (two weekdays and one weekend day) completed by caregivers, and by adolescents when appropriate. Data were collected during routine outpatient clinic appointments or home visits. The records captured all foods, drinks, low-protein products, and prescribed protein substitutes. Caregivers received written guidance with portion size examples to support accurate completion, and each record was reviewed with a specialist metabolic dietitian to ensure clarity, completeness, and consistency.

Three-day food records were used to estimate intakes of energy, protein, and selected micronutrients (calcium, iron, zinc, vitamin B_12_, and vitamin D). Information on vitamin and mineral supplement use was collected. At the time of data collection, none of the children were receiving additional vitamin or mineral supplements, outside of their prescribed protein substitutes; therefore, all reported micronutrient intakes reflect contributions from food and protein substitutes only. Dietary data were analysed using Nutritics dietary analysis software version 6.22 [32]. Entries for protein substitutes and specialised low-protein foods were reviewed for accuracy, with missing micronutrient data added to ensure accurate nutrient calculations. Micronutrient intakes were compared with UK reference nutrient intakes and expressed as a percentage of dietary reference values (%DRV) using Nutritics^®^ (Dublin, Ireland), which applies the Scientific Advisory Committee on Nutrition (SACN, 2016) values for vitamin D [33] and the Committee on Medical Aspects of Food and Nutrition Policy (COMA, 1991) [34] age- and sex-specific values for calcium, iron, zinc and vitamin B_12_. For descriptive analysis, %DRV for calcium, iron, zinc, vitamin D, and vitamin B_12_ was examined across natural protein tolerance categories defined by daily natural protein intake (<15 g/day, 15–24 g/day, 25–39 g/day, and ≥40 g/day).

### 2.3. Statistical Analysis

Descriptive statistics were used to summarise the data. Normally distributed variables are presented as means ± standard deviations (SDs), while non-normally distributed variables are reported as medians and interquartile ranges (IQRs). Normality was assessed using standard tests for distribution.

### 2.4. Ethics

The study protocol was approved by the UK Wales Research Ethics Committee (REC reference: 22/WA/0143; IRAS ID: 314071) and received institutional research and development approval from Birmingham Women’s and Children’s NHS Foundation Trust. The study was conducted in accordance with the Declaration of Helsinki, relevant UK legislation, and Good Clinical Practice guidelines. Written informed consent was obtained from caregivers, and assent was sought from children where appropriate.

## 3. Results

### 3.1. Participants

A total of 21 children (12 males; 57%) with PKU treated with sapropterin were included in the analysis. The mean age at 24 months was 12 years (range 6–19 years). Based on pre-sapropterin Phe tolerance, 14 (67%) were classified as classical PKU, 5 (24%) as mild PKU, and 2 (9%) had DHPR deficiency. All participants received once-daily sapropterin at a median dose of 20 mg/kg (range 10–20 mg/kg). Both children with DHPR deficiency were treated with 20 mg/kg/day sapropterin and received neurotransmitter precursor therapy. Two participants had a coexisting diagnosis of autism spectrum disorder. One 17-year-old was non-adherent to dietary treatment following sapropterin initiation.

### 3.2. Protein and Micronutrient Intake

Protein and micronutrient intakes for sapropterin-responsive children are shown in Table 1. Total protein intake ranged from 42 to 104 g/day, with natural protein contributing 8–66 g/day and protein substitutes providing 0–80 g/day protein equivalent. Two children did not consume protein substitutes: subject 5 because a protein substitute was no longer required and subject 20 due to non-adherence. Mean total protein intake was 75 ± 14 g/day (1.8 ± 0.8 g/kg/day), with 30 ± 16 g/day (0.7 ± 0.4 g/kg/day) from natural sources and 45 ± 21 g/day (1.1 ± 0.6 g/kg/day) from protein substitutes.

**Figure 1:**
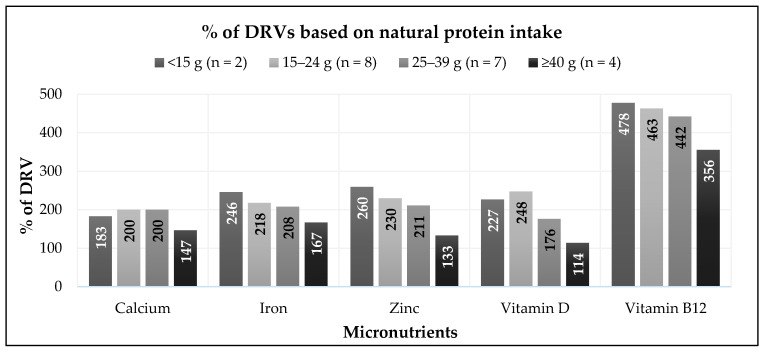
Percentage of DRVs for calcium, iron, zinc, vitamin D, and vitamin B_12_ by natural protein intake groups. (Note: Values include intake from both food and protein substitutes.)

Figure 1 shows the % DRVs for calcium, iron, zinc, vitamin D, and vitamin B_12_ across the natural protein intake groups. The median percentage DRV for most nutrients was above 100%; however, four patients had intakes below the reference values.

- Subject 5, who consumed ≥40 g/day of natural protein and had no protein equivalent intake from protein substitutes, had iron intake at 51% DRV, zinc at 90% DRV, and vitamin D at 4% DRV.
- Subject 10 (15–24 g/day natural protein group), with protein equivalent intake from protein substitutes of 60 g/day, had vitamin D intake at 85% DRV.
- Subject 14 (25–39 g/day natural protein group), with protein equivalent intake from protein substitutes of 6 g/day, had intakes below 75% DRV for iron (73%), zinc (68%), and vitamin D (51%).
- Subject 20 (≥40 g/day natural protein group), who was non-adherent with dietary management and had no protein equivalent intake from protein substitutes, had intakes below the reference values for calcium (46% DRV), iron (64% DRV), zinc (41% DRV), and vitamin D (60% DRV).

### 3.3. Micronutrient Intakes from Food Sources and Protein Substitutes

Median daily intakes of key micronutrients were predominantly supplied by protein substitutes (Table 2). Protein substitutes contributed 1100 mg/day of calcium (80%), 18.7 mg/day of iron (84%), 16.7 mg/day of zinc (87%), 20 µg/day of vitamin D (96%), and 3.7 µg/day of vitamin B_12_ (78%). Food sources provided a median of 280 mg/day of calcium (20%), 4.2 mg/day of iron (16%), 2.5 mg/day of zinc (13%), 0.5 µg/day of vitamin D (4%), and 1.3 µg/day of vitamin B_12_ (22%).

## 4. Discussion

In this secondary descriptive analysis of sapropterin-responsive children at 24 months, natural protein tolerance increased markedly but remained highly variable (8–66 g/day), while protein intake from protein substitutes ranged from 0 to 80 g/day protein equivalent. Despite enhanced natural protein intake, protein substitutes continued to provide 78–96% of total calcium, iron, zinc, vitamin D, and vitamin B_12_ intake, highlighting their central role in meeting micronutrient requirements. Although micronutrient intakes exceeded DRVs, four children had intakes below recommended amounts for calcium, iron, zinc, or vitamin D; vitamin B_12_ intake was above recommended requirements. These shortfalls occurred in three of the four children who had reduced or discontinued protein substitutes. These findings show that increased natural protein tolerance during dietary liberalisation does not ensure adequate micronutrient intake.

Notably, intakes meeting the DRV was not assured even when fortified protein substitutes were maintained. One child receiving a tablet-based Phe-free protein substitute providing 60 g/day of protein equivalent achieved only 85% of the recommended vitamin D intake, demonstrating that micronutrient gaps may persist despite apparently sufficient use of fortified preparations. This reflects an important but often overlooked issue: the micronutrient composition of protein substitutes varies between formulations, and amounts are not standardised across products [35,36]. Consequently, clinicians and dietitians must regularly review the nutritional profiles of prescribed products to ensure that changes in protein substitutes or reductions in natural food sources do not inadvertently lead to micronutrient insufficiency.

Children who reduced or discontinued protein substitutes were at particular risk of inadequate dietary intake. Three children with relatively high natural protein intakes (36–66 g/day) with reduced or no protein substitute intake had multiple micronutrient intakes below DRVs, demonstrating that increased natural protein tolerance alone does not guarantee nutritionally balanced food choices. This observation is consistent with previous evidence linking protein substitute reduction or discontinuation to lower protein and micronutrient intake [17,27,28]. Although micronutrient-dense protein substitutes are recommended when protein substitute intake is substantially reduced [17], evidence regarding adherence to these purpose-designed preparations during dietary liberalisation remains limited. These supplements provide proportionally higher micronutrient levels per unit of protein equivalent to compensate for reduced intake of conventional Phe-free protein substitutes; however, their effectiveness depends on consistent use and appropriate product selection.

Reductions in, and choice of, protein substitutes with drug treatment requires careful and proactive dietetic management. Key considerations include the nutritional quality of newly introduced foods, the consistency and sustainability of higher-protein dietary patterns, age-specific nutrient requirements, and the continued role of protein substitutes during periods of intercurrent illness [21,28,37]. Protein substitute reduction should be individualised and implemented in a structured, stepwise manner, supported by scheduled dietetic review to evaluate metabolic control, natural protein tolerance, overall nutritional adequacy, and the patient’s capacity to consistently incorporate nutrient-dense, protein-containing foods. Differences in micronutrient fortification across individual protein substitutes products must be considered when modifying prescriptions, particularly when intake is substantially reduced. Any reduction should only be initiated once a stable and sufficient intake of natural protein has been achieved in accordance with WHO/FAO/UNU safe levels of protein intake [23,38]. When protein substitute intake is reduced by more than 50%, short-term, targeted micronutrient supplementation may be required until a varied, nutrient-dense diet is reliably established and maintained.

Although increased Phe tolerance expands dietary flexibility, food choice patterns may remain limited and may be shaped by longstanding habits. Previously published data from this cohort [20] showed that higher natural protein tolerance was associated with increased intake of micronutrient-dense animal-derived foods, including dairy products, eggs, fish, and meat. However, several children continued to preferentially consume low-protein milks despite increased natural protein tolerance, reflecting food behaviour inertia commonly reported in PKU [17,25,27,39,40]. Particular attention should be given to prioritising foods containing iron, calcium, and zinc, which are consistently vulnerable during periods of reduced protein substitute use. Years of reliance on specialised low-protein foods, limited exposure to age-typical textures, and persistent perceptions of dietary risk can constrain dietary diversification, even when metabolic control allows expansion. These observations highlight that dietary expansion should not occur unsupervised once tolerance increases; rather, it requires structured and intensive dietetic oversight to ensure that rising natural protein intake is accompanied by appropriate, clinically supervised adjustment of protein substitutes. The persistence with established food choices aligns with earlier evidence of entrenched selective eating behaviours in PKU [3,41]. In addition, optimising fruit and vegetable consumption is an essential complementary strategy during liberalisation [23], as these foods provide vitamins, minerals, fibre, and phytonutrients that support overall nutritional quality [42].

Routine assessment of micronutrient intake remains essential during periods of dietary transition [23]. Although dietary intake data provide valuable contextual information, intake above DRVs does not confirm micronutrient adequacy at an individual level. Biochemical assessment therefore remains an essential component of clinical monitoring. However, reliance on biochemical markers alone may fail to detect early or subclinical insufficiency, given physiological variability, metabolic influences, and the limited sensitivity of many biomarkers to marginal deficits [43]. A combined approach, integrating dietary intake, biochemical indices, and clinical judgement remains necessary to accurately evaluate micronutrient status. Practical dietary assessment tools, such as short food records, structured recalls, or rapid dietary-screening questionnaires, can support earlier identification of emerging inadequacies and facilitate timely intervention, particularly in children whose dietary patterns and requirements change with growth.

As pharmacological treatments become increasingly integrated into routine PKU management, there is a need to reconsider when and how nutrient-dense, protein-containing foods are introduced, including prior to the initiation of drug therapy. Early, cautious inclusion of small, controlled amounts of micronutrient-rich foods (e.g., milk, yoghurt, cheese, and egg) within prescribed Phe limits may confer nutritional benefits and facilitate smoother dietary transitions if natural protein tolerance increases over time [31]. Proactive exposure to these foods may also mitigate the behavioural and sensory barriers that often complicate dietary liberalisation once pharmacological responsiveness is established.

Despite an increasing number of patients with PKU moving toward more relaxed dietary patterns, there remains a lack of educational materials specifically designed to support this transition. Existing resources are largely based on traditional models of strict dietary restriction and offer limited guidance on maintaining nutritional adequacy as natural protein tolerance increases. Consequently, families may be left without clear, practical advice on incorporating nutrient-dense, protein-containing foods, balancing natural protein with protein substitutes, or recognising early indicators of declining dietary quality during liberalisation. Updated written resources and digital tools could address these gaps by offering structured guidance on portion sizes, micronutrient-rich food choices, and strategies for sustaining dietary balance, thereby reducing the risk of micronutrient deficiencies during liberalisation. Such tools may also enhance the coordination of dietary monitoring within routine healthcare systems.

This study has several strengths, including its prospective design and detailed quantification of micronutrient contributions from both natural foods and protein substitutes. The systematic assessment of dietary intake provides valuable insight into how nutritional adequacy shifts during sapropterin-mediated liberalisation. However, several limitations should be acknowledged. First, the single-centre design may limit external validity, as dietary practices, service structures, and approaches to sapropterin management vary considerably across treatment centres and countries. The small sample size further constrains generalisability and reduces the ability to explore subgroup differences, such as variation by age, treatment duration, or degree of responsiveness to sapropterin. However, separate consideration of the two participants with DHPR deficiency did not alter the overall findings, as they were managed using the same dietary approach as the remainder of the cohort. Similarly, exclusion of the single non-adherent participant from the subgroup with natural protein tolerance ≥ 40 g/day resulted in only a modest increase in mean %DRVs for calcium, iron, zinc, vitamin D, and vitamin B_12_, with micronutrient intakes remaining above DRVs overall. These sensitivity checks indicate that the main clinical interpretation of the study remains robust. Second, dietary data were based on caregiver-reported records, which are inherently vulnerable to reporting and recall bias. Under-reporting of natural protein sources, over-estimation of portion sizes, and incomplete documentation of snacks or school meals may all influence micronutrient estimates. Third, the absence of biochemical micronutrient markers limits the ability to corroborate dietary intake with objective indicators of nutrient status.

Nevertheless, the findings clearly demonstrate that reductions in protein substitute intake during dietary protein liberalisation must be approached gradually and with structured dietetic supervision. Abrupt or poorly monitored protein substitute reductions risk compromising adequate micronutrient intake, even in children with relatively high natural protein tolerance. These results reinforce the need for proactive monitoring, tailored education, and carefully staged dietary adjustments to ensure that nutritional adequacy is maintained as treatment paradigms evolve.

## 5. Conclusions

Sapropterin treatment substantially increases natural protein tolerance in children with PKU; however, adequate nutritional intake continues to depend heavily on protein substitutes. While most sapropterin-responsive children achieved satisfactory micronutrient intake, those who reduced or discontinued protein substitutes were more likely to fall below recommended levels, particularly for calcium, iron, zinc, and vitamin D. These findings reinforce the idea that increased natural protein tolerance does not automatically translate into nutritionally balanced food choices, nor does it guarantee sufficient micronutrient intake without continued dietetic oversight. Ongoing evaluation of long-term dietary quality and micronutrient intake is therefore essential to support safe and sustainable transitions toward more flexible dietary patterns in sapropterin-treated children with PKU. Future longitudinal research that combines biochemical markers, behavioural assessments, and real-world dietary data will be critical for identifying effective approaches to support families as treatment paradigms evolve and dietary expectations change.

## Figures and Tables

**Table 1:** Protein and micronutrient intakes of sapropterin-responsive children (*n* = 21).

		Protein Intake			Micronutrient Intake				
Subject	Age at 24 Months (Year)	Total Protein (g/day, g/kg/day)	Natural Protein (g/day, g/kg/day)	PE from PS (g/day, g/kg/day)	Calcium (mg/day, DRV%)	Iron (mg/day, DRV%)	Zinc (mg/day, DRV%)	Vitamin D (µg/day, DRV%)	Vitamin B_12_ (µg/day, DRV%)
1	19	104 (1.4)	59 (0.8)	45 (0.6)	1605, 229%	28.0, 321%	18.6, 196%	25.2, 252%	6.9, 459%
2	9	72 (3.2)	32 (1.4)	40 (1.8)	1663, 302%	28.0, 322%	20.4, 291%	26.2, 261%	5.2, 515%
3	9	83 (3.2)	23 (0.9)	60 (2.3)	1508, 274%	26.9, 309%	19.2, 274%	33, 330%	6, 603%
4	13	69 (1.4)	9 (0.2)	60 (1.3)	1250, 125%	23.7, 209%	17.4, 193%	30.2, 302%	4.9, 405%
5	9	66 (1.7)	66 (1.7)	0 (0)	855, 155%	4.5, 51%	6.3, 90%	0.49, 4%	4.2, 420%
6	9	68 (1.7)	8 (0.2)	60 (1.5)	1326, 241%	24.7, 283%	22.9, 326%	15.2, 151%	5.5, 550%
7	8	76 (2.8)	31 (1.1)	45 (1.7)	1159, 210%	20.1, 231%	19.1, 273%	11.6, 115%	4, 396%
8	8	78 (2.1)	38 (1.0)	40 (1.1)	1108, 201%	17.0, 195%	13.1, 187%	20.9, 208%	5, 496%
9	9	70 (2.3)	32 (1.1)	38 (1.3)	1418, 257%	22.2, 255%	16, 229%	21.4, 214%	6.3, 625%
10	12	79 (1.6)	19 (0.4)	60 (1.2)	1509, 150%	20.0, 176%	12.3, 136%	8.6, 85%	5.6, 463%
11	6	60 (3.2)	20 (1.1)	40 (2.1)	1332, 295%	18.3, 300%	11.7, 180%	27.2, 272%	3.9, 486%
12	12	86 (1.3)	16 (0.2)	70 (1.1)	1614, 201%	29.0, 196%	21.2, 235%	36.8, 368%	6.5, 543%
13	16	61 (1.0)	21 (0.3)	40 (0.6)	1069, 133%	16.5, 111%	11.5, 163%	9.9, 98%	4.7, 310%
14	7	42 (1.0)	36 (0.9)	6 (0.1)	661, 120%	6.4, 73%	4.8, 68%	5.2, 51%	2.8, 284%
15	14	80 (2.5)	20 (0.6)	60 (1.9)	1430, 143%	27.9, 247%	20.5, 227%	27.7, 276%	5.6, 468%
16	19	85 (1.1)	40 (0.5)	45 (0.6)	1103, 157%	20.1, 231%	19.6, 205%	13.9, 139%	5.8, 385%
17	11	73 (2.1)	37 (1.1)	36 (1.0)	1425, 178%	26.6, 179%	19.5, 216%	23.9, 238%	4.6, 379%
18	14	81 (1.3)	21 (0.3)	60 (1.0)	1477, 184%	26.7, 180%	24.7, 275%	14.8, 147%	5, 412%
19	17	102 (1.5)	22 (0.3)	80 (1.2)	1763, 220%	32.9, 222%	24.4, 348%	40, 404%	6.3, 418%
20	17	55 (0.7)	55 (0.7)	0 (0)	323, 46%	9.5, 64%	2.9, 41%	6.1, 60%	2.4, 158%
21	18	81 (1.3)	31 (0.5)	50 (0.8)	1301, 130%	23.1, 204%	20.1, 211%	14.8, 147%	6, 397%
Mean ± SD	12 ± 4	75 ± 14 (1. 8 ± 0.8)	30 ± 16 (0.7 ± 0.4)	45 ± 21 (1.1 ± 0.6)	1281 ± 347 188 ± 65%	21.5 ± 7.5 208 ± 81%	16.5 ± 6.2 208 ± 79%	19.7 ± 10.9 196 ± 109%	5.1 ± 1.2 437 ± 107%

Abbreviations: DRV: dietary reference value; PE: protein equivalent; PS: protein substitute.

**Table 2:** Median (Q1, Q3) daily intake of calcium, iron, zinc, vitamin D, and vitamin B_12_ from protein substitutes and food.

Micronutrient	Total Intake	From PS	From Food	% from PS	% from Food
Calcium (mg/day)	1332 (1108, 1508)	1100 (848, 1199)	280 (186, 323)	80 (72, 84)	20 (16, 28)
Iron (mg/day)	23.1 (18.3, 26.9)	18.7 (15.9, 22)	4.2 (2.7, 4.9)	84 (72, 88)	16 (12, 28)
Zinc (mg/day)	19.1 (12.3, 20.4)	16.7 (11.1, 17.3)	2.5 (2.0, 3.1)	87 (82, 92)	13 (8, 18)
Vitamin D (µg/day)	20.9 (11.6, 27.2)	20 (11.3, 26)	0.5 (0.2, 2.5)	96 (90, 99)	4 (1, 10)
Vitamin B_12_ (µg/day)	5.2 (4.6, 6.0)	3.7 (3.3, 4.8)	1.3 (0.3, 2.1)	78 (63, 93)	22 (7, 37)

Data presented as median (Q1, Q3). Abbreviation: PS: protein substitute.

## Data Availability

The original contributions presented in this study are included in the article. Further inquiries can be directed to the corresponding author.

## Abbreviations

The following abbreviations are used in this manuscript:

BH4	Tetrahydrobiopterin
BIMDG	The British Inherited Metabolic Disease Group
COMA	The Committee on Medical Aspects of Food and Nutrition Policy
DHPR	Dihydropteridine Reductase
DRV	Dietary Reference Value
NHS	National Health Service
PAH	Phenylalanine Hydroxylase
Phe	Phenylalanine
PKU	Phenylketonuria
SACN	The Scientific Advisory Committee on Nutrition
UK	United Kingdom
